# Transmissibility and Curability of Cancer

**Published:** 1907-09

**Authors:** 


### TRANSMISSIBILITY AND CURABILITY OF CANCER.

Dr. William Seaman Bainbridge of New York City, after reviewing the evidence of concerning the theories of heredity, congenital transmission, and infectiousness or contagiousness as causal factors in the production of cancer, concludes that the hereditary and congenital transmission of cancer are subjects which require much more study before any definite conclusions can be formulated concerning them; that the contagiousness or infectiousness is far from proved; that evidence in suport of the theory is so incomplete and inconclusive that the public need not be alarmed concerning it; that the danger of accidental acquirement of cancer is far less than from typhoid fever, syhpilis or tuberculosis; that in the case of cancer there is much more danger to the attendant of septic infection, of blood poisoning from pus organisms, than from any possible acquirement of cancer; that the communication of cancer from man to man is so rare, if it really occurs at all, that it can practically be disregarded; that in cancer, as in all other disease, attention to diet, exercise, and proper hygienic surroundings, is of the utmost importance; that cancer is local in its beginning; that, when accessible, it may, in its incipiency, be removed by radical operation so perfectly that the chances are overwhelmingly in favor of its non-recurrence; that once it has advanced beyond the stage of cure, in many cases suffering may be palliated and life prolonged by surgical means; that while other methods of treatment may, in some cases, offer hope for the cancer victim, the evidence is conclusive that surgery, for operable cases, affords the surest means of cure.—Boston Medical Surgical Journal, June 27, 1907.

